# Adsorption of Saponin and Saponin–Chitosan Mixture at Water–Oil Interface and Stabilization of Oil-in-Water Emulsions

**DOI:** 10.3390/molecules30112281

**Published:** 2025-05-22

**Authors:** Katarzyna Dziza, Marcel Krzan, Ewelina Jarek, Lilianna Szyk-Warszyńska, Sonia Kudłacik-Kramarczyk, Piotr Warszyński, Eva Santini, Libero Liggieri, Francesca Ravera

**Affiliations:** 1 Unit of Genoa, Institute of Condensed Matter Chemistry and Technologies for Energy, CNR, Via De Marini 6, 16149 Genoa, Italy; kate19mail@gmail.com (K.D.); eva.santini@cnr.it (E.S.); libero.liggieri@cnr.it (L.L.); 2 Jerzy Haber Institute of Catalysis and Surface Chemistry, Polish Academy of Sciences, ul. Niezapominajek 8, 30-239 Krakow, Poland; marcel.krzan@ikifp.edu.pl (M.K.); ewelina.jarek@ikifp.edu.pl (E.J.); lilianna.szyk-warszynska@ikifp.edu.pl (L.S.-W.); sonia.kudlacik-kramarczyk@ikifp.edu.pl (S.K.-K.); piotr.warszynski@ikifp.edu.pl (P.W.)

**Keywords:** emulsion stability, interfacial tension, dilational viscoelasticity, saponin–chitosan aggregates, coalescence

## Abstract

Investigating the adsorption properties of emulsifiers at water–oil interfaces enables advances in the comprehension of the mechanisms governing emulsion ageing and stabilization. The utilization of natural compounds in emulsion formulations is increasingly relevant for those applications where it is challenging to maintain a low impact on the environment and health. We report here a study on saponin and chitosan at the interface between water and medium-chain triglycerides (MCT) oil in relation to the properties of the corresponding emulsions. Complementary experimental approaches have been adopted to investigate interfacial properties and emulsion evolution, relying on drop tensiometry, optical and confocal microscopy, and light transmission/scattering analysis. In addition, molecular dynamics simulation has been undertaken as support for the interpretation of the experimental results. The multi-technique investigation adopted here enabled a better understanding of saponin adsorption properties and of the role of chitosan in emulsion evolution. In particular, the results evidence the formation of amphiphilic saponin–chitosan complexes, which adsorb at the liquid–liquid interface and improve the stability of oil-in-water emulsions. Since the system investigated mainly consists of natural compounds, the results of this work can contribute to the development of new and efficient low-impact formulations.

## 1. Introduction

The control of emulsification and emulsion stability is of great interest to a large number of applied fields [1,2,3,4], spanning from pharmaceuticals, cosmetics, and food to the development of new nanostructured soft materials. Despite the huge number of studies dedicated to emulsions, several aspects are still worth investigating because they are relevant to today’s emulsion-based technologies and products. One of the more challenging areas of concern is the development of efficient formulations of emulsions with a low impact on the environment and health.

Many of the emulsifiers currently in use present potentially adverse effects on health and contribute to environmental pollution when spread as domestic and industrial waste. This makes the efficient and safe use of emulsifiers increasingly relevant in improving the bio-compatibility and bio-degradability of products. One possible way to address this challenge is to go in the direction of more sustainable systems, using molecules with lower toxic/pollutant effects, such as natural surfactants [5] derived from plants or vegetable wastes. On the other hand, an important aspect to consider in the optimization of emulsion formulation relies on the well-established assumption that emulsion properties, particularly the time evolution of emulsion, are strongly related to the adsorption properties of emulsifiers at water–oil interfaces [6]. These properties, in fact, strongly influence the main dynamic mechanisms governing emulsion ageing, such as drainage and drop coalescence. For example, the interfacial rheology of the adsorbed layers plays an important role in preventing the rupture of the liquid films between emulsion droplets.

The present work addresses the emulsification and emulsion stability of a water–oil system stabilized by saponin and saponin–chitosan mixtures The oil phase of such emulsions is medium-chain triglyceride (MCT).

MCT has been widely employed as a bio-compatible oil in many literature studies on emulsions [5,7,8], where some physicochemical properties are available, and in food and biomedical applications [9,10,11]. Saponin is a natural surfactant, commonly extracted from plants, whose adsorption properties at liquid interfaces have been widely investigated [12,13,14] and used in many applications, such as foam and emulsion stabilizers [2,5,7], and in biomedical applications [15]. Chitosan is a polysaccharide compound, extensively employed in bio-medical [16] and food [17,18] applications to effectively improve the stability of dispersed systems. It is also used as a bio-polymer in association with saponin [19].

Saponin and chitosan have been selected for this study because, together with the use of MCT as the oil phase, they are representative of natural, non-toxic components relevant for the development of new sustainable and bio-compatible emulsion formulations.

In our recent work [19], the surface properties of saponin and the mixed saponin–chitosan solutions were investigated in relation to their foamability and the stability of the obtained foams. In that study, a strong amphiphilicity of saponin was observed, also in the presence of chitosan, together with high values of dilational viscoelasticity of saponin and mixed saponin–chitosan solutions, which are properties particularly relevant for foam stabilization. Moreover, the synergetic effects of saponin and chitosan were evidenced regarding the long-time foam stabilization. These effects have been ascribed to the changes in bulk viscosity induced by chitosan and the fact that chitosan, even if it is not amphiphilic, indirectly influenced the adsorption dynamics of saponin.

In a successive study [20], the investigation of the same system was extended using vibrational sum frequency generation (SFG) techniques together with surface tension and dilational rheology measurements, proving the adsorption, under certain conditions, of saponin–chitosan complexes at the water-air interfaces.

In [21], the adsorption properties of saponin and other bio-compatible synthetic and natural surfactants, at the water–MCT interface, were investigated by interfacial tension and dilational rheology measurements, as well as the capability of these surfactants to form and stabilize oil-in-water emulsions. In that work, it was observed that, compared to the other surfactants (Tween80 and Citronella Glucoside), saponin was more effective in stabilizing oil in water emulsions; however, in contrast to what was observed for foams, this was not associated with higher values of dilational modulus in the investigated frequency range.

In fact, from the results obtained in [21], the capacity of saponin as an emulsion stabiliser seems to be related to the molecular features of saponin, such as its higher occupational area and ionic character, or to the high-frequency elasticity of the adsorbed layer at the liquid–liquid interface. The latter, in many cases, is not directly observed but is theoretically predicted on the basis of a fitting procedure, using appropriate models.

The present work aims at continuing our previous studies reported in [19,21]. This study is, in fact, carried out in line with that reported in [19] on saponin at the water–air interface and foams. Accordingly, here, the effect of the presence of chitosan on the adsorption of saponin at water–MCT interfaces is investigated to understand the correlation with the properties of the respective oil-in-water emulsions. Moreover, the effect of the presence of chitosan on the destabilization mechanisms of the obtained oil-in-water emulsions is investigated in detail, complementing the results on saponin obtained in [21].

## 2. Results

In this section, we report the experimental results on the interfacial properties of the mixed saponin–chitosan systems at the water–MCT interface as well as on the behavior of the respective emulsions. Such results are obtained by different experimental approaches and are discussed in order to understand the role of the adsorption properties of the emulsifiers in emulsion evolution and stabilization.

The saponin used in the present work is an extract from Quillaia saponaria, the same product used in our previous work [19,20,21]. In [19], the composition of this extract was analyzed and discussed in detail; it was found that it consists of a mixture of components with different molecular weights characterized by a rather broad distribution in range, from 1070 to 1700 g/mol.

As underlined in previous work [19,20], the solubility of chitosan in water is warranted only at quite a low pH. For this reason, the investigated mixtures were obtained by dissolving saponin and chitosan in a 10 g/L (0.17 M) solution of acetic acid. Under these conditions, as verified in [19], the pH was maintained at around 3 for all the saponin concentrations investigated. Thus, whether or not specified, the solutions containing chitosan are always prepared with acetic acid at a concentration of 0.17 M.

### 2.1. Interfacial Properties

#### 2.1.1. Adsorption Properties of Saponin with and Without Acetic Acid

The results obtained in [19] prove that, for neutral pH, saponin behaves as an ionic surfactant because its charge groups are predominantly dissociated, while, in acidic conditions obtained by adding acetic acid, the dissociation of carboxylic groups is strongly reduced, implying a non-ionic surfactant behavior with increased surface activity at the same bulk concentration.

Thus, as the adsorption properties of saponin are expected to be pH-dependent, the aqueous solutions of saponin plus acetic acid have been preliminarily investigated by measuring interfacial tension and dilational viscoelasticity, and the results are compared with those obtained with saponin dissolved in pure water.

**Figure 1:**
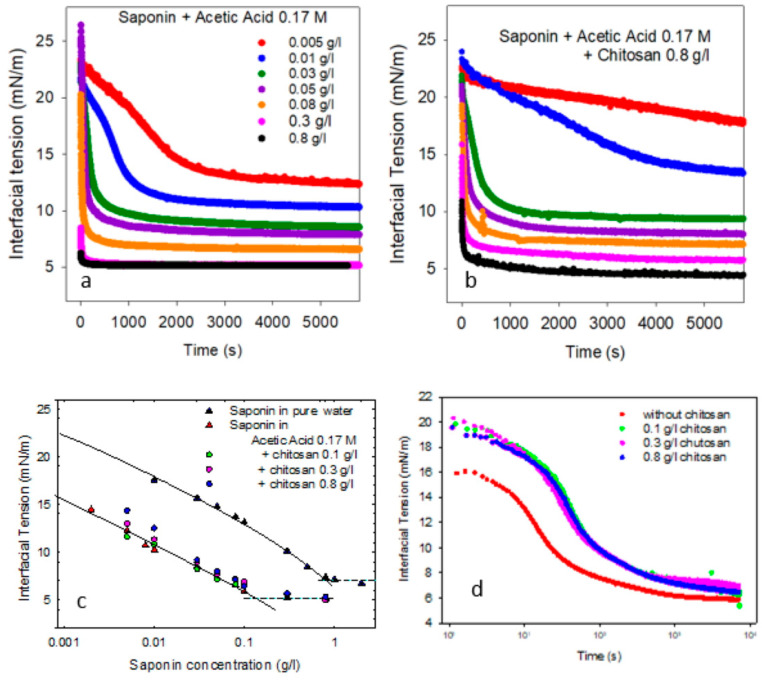
Interfacial tension versus time for saponin in acetic acid 0.17 M at water–MCT for different concentrations of saponin (**a**) and with chitosan at 0.8 g/L (**b**). Equilibrium interfacial tension versus saponin concentration (**c**) in pure water from [21] (black triangles) and in acetic acid 0.17 M (red triangles), with the corresponding theoretical curves from the reorientation model calculated with the best fit parameters reported in Table 2 (solid lines), and after the addition of chitosan at different concentrations (circles). Interfacial tension during adsorption kinetics for different content of chitosan at a given saponin concentration (0.1 g/L) (**d**), presented in logarithmic scale to better illustrate the effect of the addition of chitosan.

Figure 1a shows the trend of the interfacial tension, γ, of saponin solution in acetic acid against MCT, for different concentrations of saponin. The corresponding equilibrium values versus concentration and the resulting adsorption isotherm are reported in Figure 1c, and are compared with those obtained in [21] for saponin in pure water. Some key parameters, such as the maximum adsorption, critical micellar concentration (cmc), and the interfacial tension above the cmc, derived from the analysis of these equilibrium data, are reported in Table 1 and compared with those obtained previously for saponin adsorption at water–air interfaces. Note that, as the saponin used here consists of a mixture of components with molecular weights distributed from 1070 to 1700 g/mol, in this work, where it is necessary to express adsorption properties in significant units, we assume the value of 1650 g/mol as the average molecular weight, as in previous reference studies on saponins or mixtures of saponins [12,13,14].

The solid curves in Figure 1c are the best fit adsorption isotherms derived from the reorientation adsorption model [22]. In the framework of this model, the adsorbed molecules can assume different orientations with respect to the interface, corresponding to different occupational areas at the interface. The theoretical equation used for the fitting is as follows:

(1) $$bc=\frac{1-e^{-\frac{\Pi \omega }{RT}}}{{\left(\frac{\omega_{1}}{\omega_{2}}\right)}^{\alpha }e^{-\frac{\Pi \omega_{1}}{RT}}+e^{-\frac{\Pi \omega_{2}}{RT}}}$$

where Π = γ_0_ − γ is the equilibrium interfacial pressure and γ_0_ is the interfacial tension of the pure interface, and *ω*_1_ and *ω*_2_ are the maximum and minimum areas per mole, corresponding to the horizontal and vertical orientations, respectively. *b* and *α* are further isotherm parameters, described in detail in [22,23]. *b* expresses the surface activity of the adsorbing component; *α* is directly related to the relative weight of the limit orientation states. Moreover, according to well-established classical thermodynamic treatments of surfactant adsorption, a direct relation between the surface activity of adsorbing molecules and their molar area is assumed [23]. This condition leads to an expression for the average molar area in terms of the equilibrium interfacial tension [22,24,25], which, in the case of a two-state or reorientation model, reads as follows:

(2) $$\omega =\frac{{\omega_{1}{\left(\frac{\omega_{1}}{\omega_{2}}\right)}^{\alpha }e}^{-\frac{\Pi \left(\omega_{1}-\omega_{2}\right)}{RT}}+\omega_{2}}{1+{{\left(\frac{\omega_{1}}{\omega_{2}}\right)}^{\alpha }e}^{-\frac{\Pi \left(\omega_{1}-\omega_{2}\right)}{RT}}}$$

Thus, the average molar area, *ω*, decreases with the decreasing of the equilibrium interfacial tension, corresponding to the advancement of the surfactant adsorption, and approaches the value associated to the vertical orientation with respect to the interface.

More sophisticated thermodynamic models have been recently developed for re-orientable surfactants adsorbing at liquid–air interfaces, taking into account different molecular reorientation modes [26]. However, the model assumed here appears to be appropriate for describing the adsorption of saponin at the water–MCT interface, independently of the presence of acetic acid. The use of Equation (2), which is a limiting case of more general theoretical expressions, provides physically realistic values for the thermodynamic parameters, enabling a coherent interpretation of the acquired data.

Based on these results, it is clear that saponin, when dissolved in acetic acid, presents higher surface activity and lower cmc. The best fit value found for the isotherm parameter b (Table 2) is one order of magnitude lower compared with that found for pure water. As in the water–air interface, this reduction is the result of the suppression of the charge of saponin at a low pH, which makes it predominantly behave as non-ionic surfactants. Additionally, at that pH, the carboxylic groups of fatty acids of MCT at the interface with water are protonated; therefore, the repulsive electrostatic interaction between saponin and the MCT interface is diminished.

The other isotherm parameters are of the same order of magnitude, even if ω_2_ is slightly reduced in acetic acid. This effect is explainable considering that, being adsorbed saponin predominantly uncharged, as expected at a low pH, the reduction of repulsive forces between the adsorbed molecules results in a lower value of the area per adsorbed molecules.

#### 2.1.2. Partitioning

To complement this study on the adsorption properties of saponin, the possibility of the transfer into the oil phase was investigated. As underlined in many previous studies [6,25], the surfactant partitioning in liquid–liquid systems is an important aspect that must be taken into account because it can affect the dynamics of the interfacial layers and modify the morphology and stability of the corresponding emulsions.

For saponin in the water–MCT system, different behaviors are expected depending on whether saponin is dissolved in pure water or in acetic acid solutions. In the first case, as saponin is predominantly dissociated, the transfer across the water–oil interface can be neglected. On the other hand, at a low pH, when saponin is essentially a non-ionic surfactant, such partitioning can be relevant.

Methods to quantitatively evaluate the partition coefficient; that is, the ratio between the surfactant concentrations in oil and in water at equilibrium, have been developed and widely used [25,27]. However, the occurrence of the surfactant transfer across the water–oil interface can be preliminarily checked by an effective and easy method relying on the comparison of the interfacial tension trends obtained with two different configurations: a drop of oil immersed in the aqueous surfactant solution and a pendant drop of solution in an initially surfactant oil-free phase. In the case of surfactant transfer, the second configuration provides higher values of interfacial tension, due to the impoverishment of the drop phase because of the much higher volume of the initially pure external phase, or even, when the partitioning is very favored, a typical minimum trend [25].

As shown in Figure S1 of the Supplementary Materials (SM), for saponin dissolved in pure water, the trends of interfacial tension are coincident, while, in the presence of acetic acid, the interfacial tension in the second configuration remains slightly higher during adsorption. Therefore, we can conclude that, while in the first case the transfer of surfactant is not relevant, in acetic acid, a slight surfactant transfer can be hypothesized; however, it does not appreciably influence the adsorption dynamics of the system.

#### 2.1.3. Effect of Chitosan on Interfacial Properties

The interfacial properties of the mixed saponin–chitosan solutions against MCT have been investigated for different concentrations of chitosan, ranging from 0.1 to 0.8 g/L. As an example, Figure 1b shows the trends of the dynamic interfacial tension of mixed solutions containing chitosan at 0.8 g/L and different concentrations of saponin. The corresponding equilibrium values versus saponin concentration are reported in Figure 1c, in order to evidence the effect of the presence of chitosan on the saponin adsorption isotherm.

From these results, it is evident that the adsorption process in the presence of chitosan is, in general, slower and, depending on the composition, produces higher equilibrium values of interfacial tension. This is especially true for concentrations of chitosan around 0.3 g/L and higher. In fact, from Figure 1c, it is clear that, while for 0.1 g/L of chitosan, the equilibrium interfacial tension versus saponin concentration is essentially unchanged, for higher concentrations of chitosan, the equilibrium interfacial tension tends to be increasingly larger while reducing the saponin concentration. Moreover, as shown in Figure 1d for 0.1 g/L of saponin, the presence of chitosan tends to slow down the kinetic process, also in cases where the final equilibrium interfacial tension is not affected. This behavior may be explained considering that chitosan, under low-pH conditions, is essentially a cationic polymer, well dissolved in water and without any particular amphiphilicity at the water–oil interface; therefore, its effect on the interfacial tension is essentially due to the interaction with saponin and, in particular, with the small fraction of dissociated molecules of saponin. That implies the formation of non-amphiphilic chitosan–saponin complexes, which impoverishes the surfactant content in the aqueous solution. Such depletion is more important at low-saponin concentrations than at high ones, where the amphiphilicity of saponin in the complex with the chitosan molecule can prevail.

A second effect that may contribute to the slowing down of the saponin adsorption kinetics is the increase in bulk viscosity due to the presence of chitosan. Some data on the increase of the viscosity due to the presence of chitosan are reported in [19], with the composition of the aqueous phase in the same range as the present study. In that study, it was found that, with a chitosan concentration of 0.8 g/L, the viscosity can reach a value of 3.092 ± 0.004 mm^2^/s. This increase in viscosity may slow down the bulk diffusion of saponin, inducing consequentially longer equilibration times of adsorption. Similar effects were observed in [19] in relation to the adsorption at water–air interfaces and attributed, also in that case, to the slowing down of the bulk diffusion process.

#### 2.1.4. Dilational Rheology

**Figure 2:**
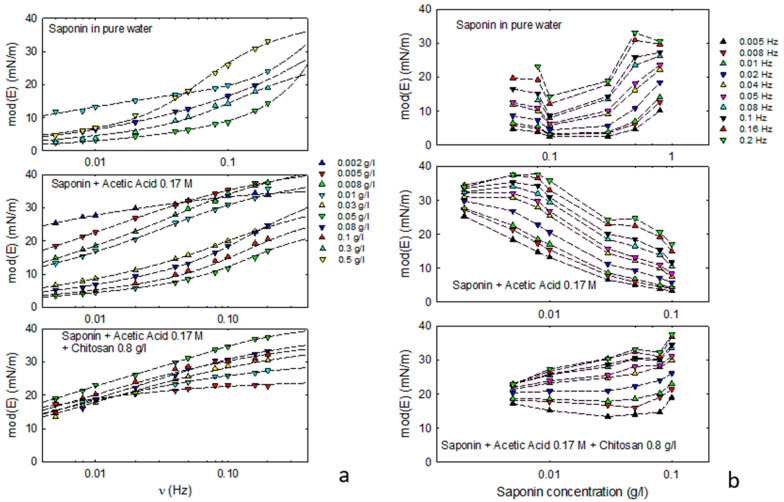
Modulus of the dilational viscoelasticity versus frequency (**a**) for saponin in pure water (data from [21]), saponin in acetic acid without chitosan and with chitosan at 0.8 g/L. Dashed lines are the best fit theoretical curves from the extended Lucassen–Van der Tempel model (Equation (3)). The same values of mod(E) plotted versus saponin concentration (**b**) to better evidence the dependence on the composition of the aqueous phase.

To obtain more information on the adsorption dynamics of these composite systems, interfacial rheology studies have been carried out against the composition of the aqueous phase. In particular, the frequency dependence of dilational viscoelasticity, *E*(*ν*), were measured by varying the saponin bulk concentration and evidencing the effects of acetic acid and chitosan. Figure 2 reports the modulus of the viscoelasticity, mod(E), as obtained by the oscillating drop method in the case of saponin dissolved in acetic acid solution, with and without chitosan, compared to mod(E) values obtained in [21] for pure water. The frequency dependence of these data (Figure 2a) shows that, for all the investigated compositions, the interfacial layer presents a viscoelastic behavior in the investigated frequency range coherent with the coexistence of the bulk diffusion and a further re-arrangement surface process.

The obtained mod(E) data are, in fact, well-fitted by the theoretical curves calculated by the following extended Lucassen–Van der Tempel equation [24,28]:

(3) $$E=\left[\frac{E_{1}+E_{0}\lambda^{2}}{1+\lambda^{2}}+\left(E_{1}-E_{0}\right)\frac{i\lambda }{1+\lambda^{2}}\right]\frac{1+\xi +i\xi }{1+2\xi +2\xi^{2}}$$

where $\xi =\sqrt{\nu_{D}/2\nu }$, $\lambda =\nu_{k}/\nu$, $\nu_{D}=D{\left(\frac{\partial c}{\partial \Gamma }\right)}^{2}$ and *ν_k_* are the characteristic frequencies of diffusion and the interfacial kinetic process, respectively. $E_{0}=\frac{d\gamma }{dln\Gamma }$ is the thermodynamic Gibbs elasticity and *E*_1_ is the high frequency limit of *E.*

Equation (3) has been widely used in many studies dealing with common soluble surfactants where adsorption is not only governed by bulk diffusion, but also by further relaxation interfacial processes [29]. The classical Lucassen–Van der Tempel equation expression is as follows:

(4) $$E=E_{0}\frac{1+\xi +i\xi }{1+2\xi +2\xi^{2}}$$

It is found as the limit case of Equation (3) for λ→+∞, which corresponds to the instantaneous equilibration of the relaxation process, or diffusion-controlled adsorption.

For the systems investigated here, as in [21], the re-arrangement process occurring in the adsorption layer in response to surface perturbation is expected to be strictly related to the variation of the average molar area under dynamic conditions, likely due to the reorientation of the adsorbed molecules.

Figure 2a shows that the observed frequency dependence of the viscoelasticity is in good agreement with the extended Lucassen–Van der Tempel model. The best fit parameters obtained and, in particular, the characteristic frequencies associated with the occurring dynamic processes (diffusion and surface re-organization), provide information on the mechanisms governing the adsorption dynamics. Figure S2 of the Supplementary Materials reports the best fit values of some key parameters, such as the characteristic frequencies of the diffusion and interfacial processes and the high-frequency limit of the dilational elasticity.

For saponin plus acetic acid, the characteristic frequency of the surface re-arrangement is found to vary from 0.03 Hz to 0.14 Hz, slightly increasing with the concentration of saponin above 0.05 g/L. The characteristic frequency of the diffusional transport process was found to be in the 0.001–0.2 Hz range, increasing with concentration.

In the presence of chitosan at 0.8 g/L, the re-arrangement at the interface does not appear relevant. The best fit theoretical curves correspond, in fact, to the limit case of the Lucassen–Van der Tempel model (Equation (4)), meaning that the diffusion transport is the main process governing the rheological response. In this case, the diffusional characteristic frequency is around 10^−3^–10^−2^ Hz, reduced of about one order of magnitude with respect to the previous cases without chitosan, but with the same increasing trend versus concentration.

These results can be interpreted considering the formation of saponin–chitosan complexes in the aqueous phase, where adsorption at the interface is controlled by a diffusion process slower than that governing the adsorption of saponin molecules and for which the surface reorientation kinetics are not significant.

The trend of the viscoelasticity against frequency is also important because it is related to the capacity of adsorbed components to hinder the phenomenon of droplet coalescence in emulsions. Higher values of mod(E), in fact, due to Marangoni effects, tend to hamper the thinning of the liquid films between droplets, preventing their breakage. As coalescence has a major role in emulsion destabilization [6], the results presented in this section are expected to be correlated with the long-time stability of the respective emulsions.

In this area, to know the trend of the elasticity in a wide frequency range is important. When this range is rather limited, as in the present work, fitting appropriate theoretical curves to the experimental rheological data enables the behavior at a high frequency to be accessed. In particular, fitting Equation (3) to the obtained E(ν) data provides the high-frequency limit of the dilational elasticity as a best fit parameter. From this kind of analysis, it was found that, while in the case of saponin in pure water [21], the high-frequency elasticity from the best fit curves reaches rather high values, of the order of 50–100 mN/m, this is not true for saponin in acetic acid, for which the limit values of the elasticity at high frequency are found to be lower than 50 mN/m (see Figure S2 in Supplementary Materials). In the presence of chitosan, the high frequency values of the elasticity are slightly increasing with the concentration but are always below those of saponin in pure water.

Figure 2b shows the values of mod(E) versus the composition of the aqueous phase in the investigated frequency range. From these data, it is evident that maximum values of mod(E) are found at different concentrations of saponin, depending on the presence of acetic acid and chitosan. More specifically, while for saponin in pure water, mod(E) increases with concentration and seems to reach a maximum at a concentration close to the cmc, in the presence of acetic acid, a maximum appears at concentrations almost two orders of magnitude lower. Increasing the concentration above that value, mod(E) tends to decrease. Adding chitosan, the trend of mod(E) is to slightly increase with the concentration, and it maintains at rather high values in almost all the concentration ranges investigated.

The correlations of these results with the long-time stability of the respective emulsions are discussed in the following sections.

### 2.2. Results from Molecular Simulation

The simulations were performed for four scenarios: fully charged chitosan chain (charge +24 e) and uncharged saponin (pH < 2); weakly charged chitosan (charge +7 e) and uncharged saponin; weakly charged chitosan (charge +7 e) and charged saponin (−1 e); and moderately charged chitosan (+15 e) and charged saponin. The case of uncharged chitosan was disregarded as, in such conditions, it was insoluble in water. The specific charge states were selected to reflect weakly, moderately, and fully charged chitosan. It is problematic to directly correlate these states with the pH of the solution. Because the pKa of chitosan depends on the molecular weight and deacetylation degree of chitosan (10.1016/j.carbpol.2006.01.001), it ranges from 6.0 to 6.9; thus, the respective pH conditions are approximately (6.5, 5.0, <4.0).

**Figure 3:**
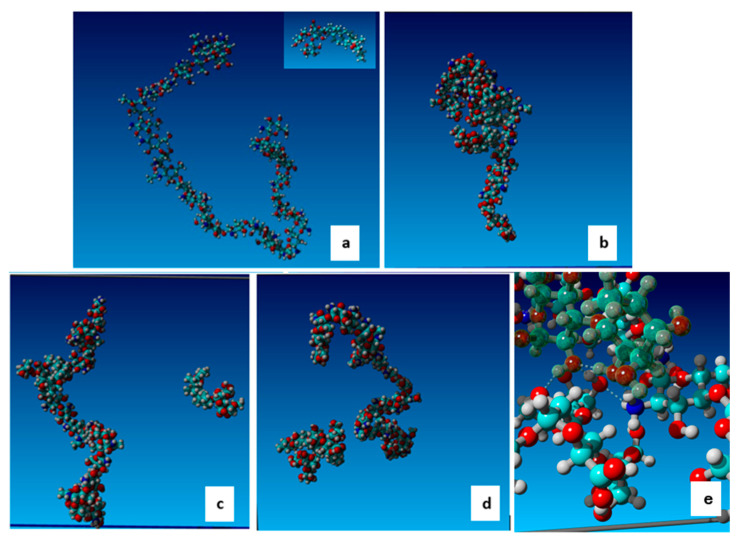
Structures of chitosan and saponin used for the molecular dynamics simulations (**a**) and snapshots from the simulations illustrating aggregation states: aggregate of chitosan and two saponins (**b**); aggregate of chitosan and one saponin with second saponin detached (**c**); chitosan with detached two-saponin aggregate (**d**); close-up showing the area of interaction (hydrogen bonds marked by dashed lines) (saponin molecule marked by grayish colors) (**e**).

Each run for 200 nm was repeated three times for different starting configurations. Figure 3 illustrates exemplary snapshots from the simulations showing the aggregation of chitosan, whereas Figures S3 and S4 of the Supplementary Materials report the exemplary time evolution during simulations of the distance between chitosan and saponin molecules, hydrogen bond energy, and hydrophobic interactions. The minimum (between closest atoms) distance between chitosan and saponins, number of hydrogen bonds, hydrogen bond energy, and hydrophobic interactions were monitored every 0.05 ns and averaged over 5 ns. Chitosan and saponin were considered as forming an aggregate if the minimum distance was below 0.25 nm for at least 5 ns. The probability of forming aggregate was determined as the ratio of time that chitosan and saponin stayed in the aggregate state to the overall time of simulations. The average hydrogen bond energy was calculated as a mean energy in the aggregated state. The results obtained, reported in Table 3, indicate that both electrostatic interactions and hydrogen bonding play a part in the formation of saponin–chitosan aggregates. While electrostatic attraction between cationic chitosan and anionic saponin determines the stability of aggregates, for uncharged saponin and moderately charged chitosan, hydrogen bonding is sufficient to sustain the aggregation of some fraction of saponin. The apparent increase of the hydrogen bond energy with electrostatic interactions results from the closer distance between interacting species and the longer lifetime of the aggregates, resulting in the maximization of hydrogen bonds. According to the expectations, the hydrophobic interactions do not play any role in chitosan–saponin aggregation as their average value is below 5, which is considered a threshold value for possible “hydrophobic bonding”; however, they play a role in the aggregation of saponin in uncharged conditions. The possibility of forming aggregates between chitosan and saponin solely due to hydrogen bonds; that is, when the saponin is uncharged under low pH conditions, explains the effect of chitosan on surface tension kinetics and surface rheology.

### 2.3. Emulsion Properties

**Figure 4:**
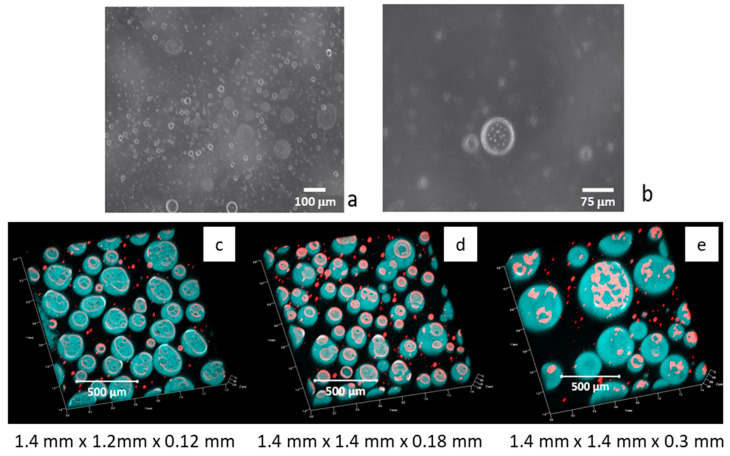
Image of a freshly formed emulsion obtained with 0.1 g/L of saponin plus chitosan 0.05 g/L in acetic acid (diluted 1:10), taken by a DVM6-M Microscope (**a**); magnified detail of the emulsion image showing the double emulsification (**b**). Evidence of double emulsification by confocal microscopy, where Coumarin 6 (blue one) and Rhodamine B (red one) were used as dyes in the oil and aqueous phases, respectively, for emulsions containing 0.1 g/L saponin in 1% of acetic acid and chitosan at different concentrations: 0.1 g/L (**c**), 0.3 g/L (**d**), and 0.8 g/L (**e**).

The first analysis of emulsion behavior has been carried out by monitoring the volume of the emulsified phase, referred to as the total volume of the liquid–liquid system, V_rel_ = V_emul_/V_tot_, after the emulsion formation. For this first study, as well as for all the other emulsion investigations reported below, the method of emulsification adopted is the double-syringe method, described in Section 4.2. In all cases, the emulsification method adopted provides the total emulsification of the liquid–liquid system so that, for all the systems investigated, V_rel_ = 1 at the initial monitoring time. The morphology of the obtained emulsions, just after the formation, has been checked by microscope visualization. By this analysis, even if rather qualitative, it was possible to verify that, in all cases, the used emulsification method provides oil-in-water emulsions with droplet sizes of the same order as those found in [21] for saponin dissolved in pure water; that is, 5–10 μm, and with the presence of some bigger droplets in presence of chitosan, which can reach diameters of about 50 μm. A typical microscope image obtained with saponin and chitosan, of a freshly formed emulsion diluted 1:10 in water, is reported in Figure 4 for visualization. At closer observation, the presence of very small droplets inside the bigger ones is observed. This leads us to hypothesizing the onset of double emulsification, which will be discussed below.

**Figure 5:**
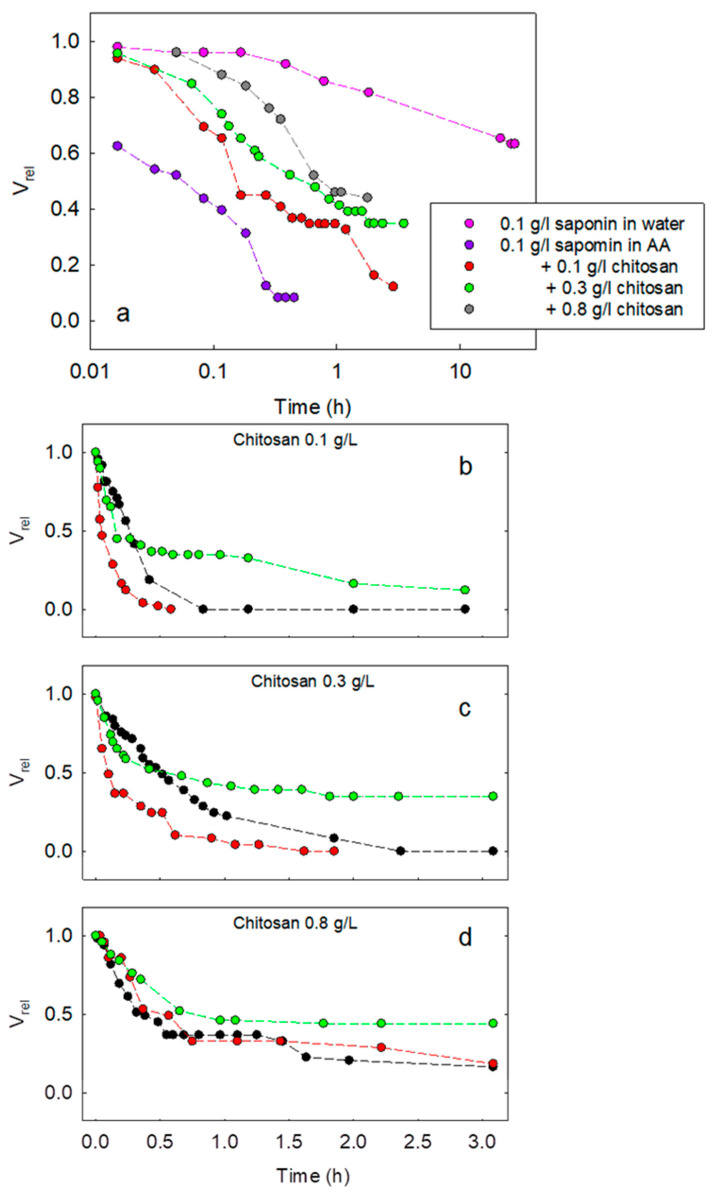
Time evolution of the emulsion volume for different compositions of the aqueous phase: saponin at 0.1 g/L in pure water, in acetic acid, and for different concentration of chitosan (**a**), and saponin at 0.01 g/L (black), 0.05 g/L (red), and 0.1 g/L (green) with chitosan at 0. 1 g/L (**b**), 0.3 g/L (**c**), and 0.8 g/L (**d**).

Figure 5 reports the main results obtained on the variation of V_rel_ with time for different compositions of the aqueous phase of the emulsions during their destabilization. Figure 5a illustrates the effect of pH, or the presence of acetic acid, on the evolution of the emulsion as well as that of the content of chitosan. It is evident that, for saponin at 0.1 g/L with acetic acid, emulsions destabilize much earlier than those obtained with saponin in pure water. Note that the cases for saponin concentration below 0.1 g/L in acetic acid without chitosan are not reported in the figure because emulsions separate immediately. This behavior can be ascribed to the fact that saponin in pure water presents higher values of dilational viscoelasticity modulus at a high frequency, which likely favours the prevention of droplet coalescence due to the Marangoni effect. Moreover, the ionic character of saponin in pure water induces repulsive interactions between the adsorbed layers at MCT droplet surfaces. Such electrostatic repulsions play an important role in hindering coalescence [6]. In the presence of acetic acid, these two stabilizing effects are not relevant. In fact, the adsorbed layer can be assumed to be neutral because of saponin’s non-ionic character, which, at low pH, is predominantly not dissociated, and dilational viscoelasticity does not increase appreciably with the frequency.

The effect of chitosan is, instead, to increase the lifetime of the emulsions. This improvement in emulsion stability, which appears to be proportional to the chitosan concentration, is correlated with the trend of dilational viscoelasticity. Mod(E) presents, in fact, values slightly higher than those for saponin at the same concentration solely in acetic acid. Steric effects should be also taken into consideration due to the expected formation of chitosan–saponin complexes adsorbing at the aqueous phase–MCT interface. Moreover, in these cases, the ionic character of these complexes, due to chitosan, may increase the stability against coalescence by electrostatic repulsion.

In Figure 5b–d, the trends of V_rel_, obtained for fixed chitosan concentration and varying saponin concentration from 0.01 g/L to 0.1 g/L, are compared. It is interesting to note that, apart from the chitosan concentration of 0.8 g/L, in which increasing the saponin concentration increases the emulsion lifetime, for the lower chitosan concentrations, this proportionality with the saponin concentration is not observed. This behavior is coherent with a scenario characterized by an interplay between the two main mechanisms contrasting droplet coalescence: the fluid dynamic effect governed by dilational rheology and electrostatic repulsion between the adsorbed layers. Depending on the composition of the aqueous phase, one of the effects can be predominant over the other. For a rather low concentration of chitosan, in fact, the electrostatic and steric effects are possibly ineffective in counteracting coalescence. At the same time, mod(E) is expected to have the same decreasing trend with saponin concentration as in the case of saponin in acetic acid. Thus, while the interfacial rheology may have a role for saponin at 0.01 g/L, it becomes less effective for higher saponin concentrations. Meanwhile, when further increasing saponin concentration, the formation of charged amphiphilic complexes with chitosan is favored. Such complexes adsorb at the interfaces and hamper coalescence.

**Figure 6:**
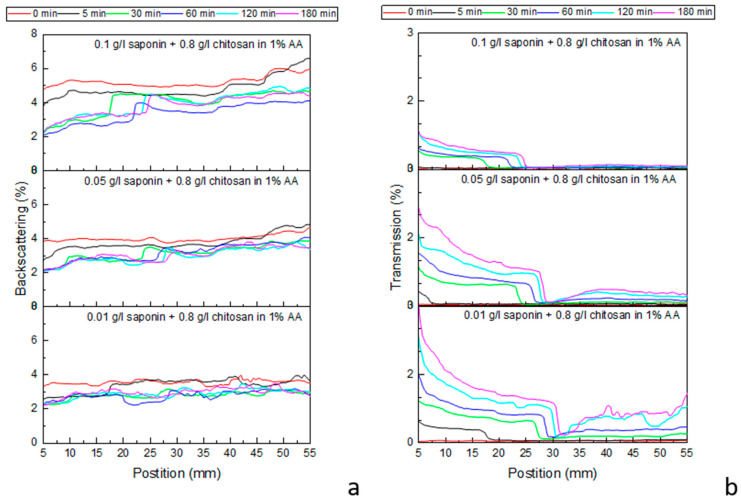
Backscattering (**a**) and transmission (**b**) intensity profiles acquired by a Multiscan MS 20. Emulsions obtained with 0.8 g/L chitosan and various saponin concentrations (0.01 g/L, 0.05 g/L, and 0.1 g/L).

**Figure 7:**
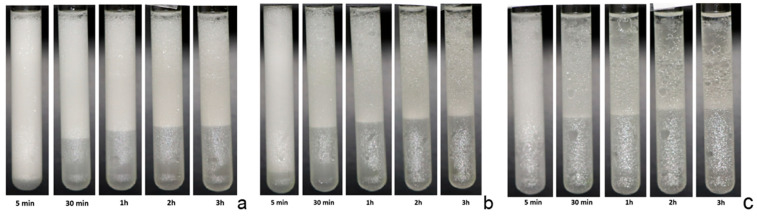
Photos of the emulsion in the Multiscan 20 cell. Emulsions obtained with 0.8 g/L chitosan and various saponin concentrations: 0.1 g/L (**a**), 0.05 g/L (**b**), and 0.01 g/L (**c**).

To go into more detail about the effect of chitosan and, in general, the dependence of the emulsion stability on the emulsion composition, the same systems reported in Figure 4 have been investigated by multiple light scattering measurements, according to the method reported in Section 4.2. Figure 6 shows the profiles recorded by a Multiscan MS 20 during the destabilization of emulsions, obtained with chitosan at 0.8 g/L and different saponin concentrations (compositions corresponding to Figure 5d) within 3 h of their generation. The results for lower concentrations of chitosan are reported in Supplementary Materials (Figures S5 and S6). Additional information on the evolution and stability of the emulsion can be obtained from the photos of the samples analyzed in the Multiscan apparatus (Figure 7, Figures S7 and S8). These photos provide a qualitative monitoring of the emulsion changes during their ageing, which is important in validating the spectra interpretation. Analysis of the acquired spectra confirms the emulsion behavior reported in Figure 5, providing further interesting details about the development of the investigated emulsions.

For all studied cases, the recorded transmission profiles prove that the evolution of the emulsions is characterized by an evident separation into two phases after a short time from the introduction of the emulsion into the cell. The spectra acquired immediately after the generation are, in fact, coherent with the presence of one emulsion initially occupying the whole measurement cell, while the spectra acquired after 5 min demonstrate the separation of the system into two different phases: an emulsion with droplet sizes of the order of some microns in the superior part of the cell (impenetrable to the Multiscan NIR radiation wavelength), and an initially wholly opaque phase in the lower part of the cell. This latter phase is expected to also be an emulsified phase that, as time progresses, becomes more and more transparent to light radiation as oil drops tend to flow out under the influence of buoyancy. In accordance with the previously carried out microscope observation, we found that all the emulsions obtained in this work are oil-in-water emulsions; therefore, we can assume that the emulsified phases observed during the Multiscan MS 20 measurements are oil-in-water emulsions as well.

Note that, in the previous more qualitative analysis of emulsion ageing, the results of which are reported in Figure 5, this lower phase was not recognized as an emulsified phase but considered from the beginning to be an aqueous phase, separated from the emulsions due to their destabilization. After 30 min from the formation, the boundary between the two detected phases is at a 25–30 mm level and only slightly increases during succeeding evolution. The backscattering spectra confirm this dynamic behavior, even if only qualitatively, due to the low percentage of reflection radiation. The images reported in Figure 7 provide further confirmation of this behavior.

With a more rigorous analysis of the spectra, also confirmed by the emulsion images, one can assert that a boundary phase is formed between these two emulsions, which are completely impermeable to radiation, as evidenced in Figure 6b (transmission is equal to zero), and slightly increase in thickness over time. The formation of this further layer between the two principal emulsified phases is most likely due to the chemical or physicochemical processes occurring in these areas under the dynamic conditions of the early destabilization process, such as aggregation, coagulation, or gelation processes, as well as involving the components of the oil fraction.

A common behavior observed in all the emulsions investigated here, with different concentrations of saponin and chitosan in the period of up to 180 min from generation, consists of a noticeable change of the emulsions in the lower part of the cell from the beginning of the acquisition, while the emulsions composed of bigger droplets in the upper part seem to be more stable, especially those obtained with saponin at 0.1 g/L (see Figure 6, Figures S5 and S6). This can be explained by assuming that the smaller droplets in the lower emulsion layer aggregate and/or coalesce, and that the resulting bigger droplets or aggregates tend to transfer into the boundary phase. This implies that part of the aqueous phase is displaced downwards, increasing the content of water in the emulsion in the lower part of the cell, which results in the observed changes in the transmission curves. Meanwhile, the upper emulsion becomes increasingly rich in the oil phase but with a longer destabilization time.

In summary, we can say that the obtained spectra and their variations in time are coherent with a scenario where an initially uniform emulsion separates, mainly driven by the buoyancy effect, into two oil-in-water emulsions: the lower part of the cell contains smaller (micrometric) droplets; whatever coalesced into bigger droplets under the effect of gravity tend to rise towards the emulsion in the upper part of the cell. The upper emulsion becomes more and more dense; its slower destabilization is driven by the coalescence of oil drops.

To complement the results reported so far, the morphology of the emulsions was analyzed by confocal microscopy, using Coumarin 6 and Rhodamine B as dyes dissolved in the MCT oil and aqueous phase, respectively. The concentrations of dyes adopted in this study were optimized by interfacial tension measurements and optical observations. The concentration of Rhodamine B in aqueous solution was 3 × 10^−7^ M, while the MCT solution of Coumarin 6 was obtained by dissolving 10 μL of a saturated solution of Coumarin 6 in 1 mL of MCT. At these concentrations, the measurements of the interfacial tension indicated negligible effects on the adsorption properties of emulsifiers (see Table S1). Additional emulsification tests also demonstrated the negligible influence of these concentrations on emulsion behavior. The emulsions for the confocal analysis were prepared using the double-syringe technique, as described in the previous section, and micrographs were taken for freshly formed emulsions diluted with water 1:10. In Figure 4c–e, the confocal microscopy images from emulsions obtained with saponin at 0.1 g/L and chitosan are reported for different concentrations.

These images evidence the presence of a certain amount of aqueous phases inside the oil droplets, confirming the onset of double emulsification that was already observed by optical microscopy. Moreover, this phenomenon appears to become more important with the increasing of the chitosan concentration, which also induces an increase in the oil droplet size.

## 3. Discussion

The results reported above on the properties of the composite saponin–chitosan system at the water–MCT interface, as well as of the corresponding emulsions, find a coherent interpretation assuming the formation of chitosan–saponin complexes in the aqueous phase, which was previously hypothesized in [20] by zeta potential measurements versus chitosan concentration.

The formation of these complexes is expected to be driven mainly by the electrostatic interaction between chitosan, which is essentially a cationic macromolecule, and the dissociated part of saponin present in the acetic acid containing the aqueous phase. In fact, even when, under low pH conditions, the portion of dissociated molecules is rather low, such a percentage is re-stablished once these molecules are captured by chitosan to maintain the balance between the dissociated and not-dissociated molecules determined by the pH value.

The results of molecular dynamics calculation indicate that chitosan–saponin aggregates are mainly formed and stabilized by the electrostatic interactions, but they can be rendered reasonably stable by hydrogen bonds in the case of weakly charged chitosan and uncharged saponin; therefore, the aggregation can be observed even at a low pH when the saponin is neutralized, provided the charge of chitosan is not too high. For fully charged chitosan, although the number of water molecules with hydrogen bonding is the same as for the weakly charged one (on the average 150 in our case), the prevalence of the hydrogen bond donor sites presumably makes the hydration water structure less favorable for the formation of chitosan–saponin aggregate.

In [20], the SFG measurements also indicated the presence of chitosan at the water–air interface, supporting the hypothesis of a certain degree of amphiphilicity in the aggregates. The hydrophilic part of such aggregates is expected to be composed of the positively charged chitosan macromolecule, while the hydrophobic part is due to the aglycone structure of the saponin molecules aggregated to chitosan.

From the interfacial tension measurements, the amphiphilicity of such complexes does not appear to be relevant. The principal effect of the presence of chitosan is, in fact, that of reducing the adsorption of saponin, especially at low concentrations (Figure 1), and slowing down adsorption kinetics (Figure 1d). This behavior can be explained by considering bulk effects solely, such as the capture of a part of free saponin molecules by chitosan and the increase of the bulk viscosity, which reduces the rate of the diffusional transfer to the interface.

However, a deeper analysis of saponin adsorption properties, carried out by dilational rheology measurements, indicated that, in the presence of chitosan, the mechanism of adsorption is essentially diffusion controlled, with characteristic time coherent with the hypothesis of adsorbing complexes without any apparent interfacial relaxation process. Different results are found in cases of saponin alone, where an interfacial process seems to be relevant and likely related to the molecular reorientation of surfactants at the interface.

Thus, the improvement of the stability of oil-in-water emulsions in the presence of chitosan (Figure 5) may be explained by considering the adsorption of amphiphilic saponin–chitosan complexes, which contributes to the formation of relatively rigid adsorbed layers at the liquid–liquid interface, characterized by a relevant positive charge.

In practice, while at a low saponin concentration, the effect of the presence of chitosan can be that of reducing saponin adsorption because the formed complexes are predominantly hydrophilic, at higher saponin concentrations, these complexes are amphiphilic and, once adsorbed at the liquid–liquid interface, can form layers very effective in stabilizing emulsions against coalescence, both for electrostatic repulsion and steric effects.

The formation of interfacial layers with these characteristics at the water–MCT interface also provides a justification for the double-emulsification process observed by confocal microscopy (Figure 4). The formation of double emulsions may also be due to the presence of minute quantities of free fatty acids C8 and C10 in the MCT oil. They are relatively soluble in water and can stabilize small droplets in the water-in-oil phase due to their hydrophilic heads containing a carboxylic group. This effect does not completely explain the dependence of double emulsification on the presence of chitosan. On the other hand, under the dynamic conditions associated with the emulsification process, small droplets of water completely covered by saponin–chitosan complexes may be transferred in the oil phase, forming a water-in-oil emulsion that is very stable for steric reasons. This transfer is expected to be favored by the increase in the concentration of chitosan, which should also increase the size of the transferred water droplets.

## 4. Materials and Methods

### 4.1. Materials

The medium-chain triglyceride (MCT) was Miglyol 812N, purchased from IOI OLEO (Hamburg, Germany) and utilized without further purification. Miglyol 812N contains about 95% of triglycerides between C8 (50–65%) and C10 (30–45%) vegetable fatty acids, while the remaining 5% is composed of lighter or heavier triglycerides and free fatty acid, with a maximum acid value of 0.2 mg KOH/g, according to the producer. Interfacial tension measurements against water enabled the exclusion of significant surface-active impurities in the oil. A constant value of 25.4 ± 0.2 mN/m at 20 °C was found, in agreement with the literature [2,5,7,8].

Saponin was reagent grade, purchased from VWR (cat. no. VWRV0163, Radnor, PA, USA) and utilised as supplied. The same product was already used and analyzed in more detail in [19,20,21].

Low molecular weight (50–190 kDa) chitosan (β-(1-4)-linked 2-amino-2-deoxy-D-glucose) was purchased from NANOSHEL in the form of high-density powder with a purity degree of 99.9% and used as received. Aqueous solutions of chitosan were prepared by dissolving the powder in 10 g/L (0.17 M) of acetic acid solution under overnight continuous magnetic bar stirring. Acetic acid was purchased from Sigma-Aldrich (Saint Louis, MO, USA) with a purity grade higher than 99.7% and used without further purifications.

The dyes used for the confocal microscopy analysis of emulsions were Coumarin 6, a hydrophobic fluorescent dye that emits in solid and solution state, purchased from Sigma-Aldrich (cat. no. 442631-1g; 98% purity), and Rhodamine B, a water-soluble fluorescent dye purchased from POCh (cat. no. 764/40/14; pure for analysis).

The water used for the preparation of all the solutions was produced by a Millipore (Elix + Milli-Q) purification system, with a resistivity higher than 18 MΩ∙cm, a surface tension of 72.5 ± 0.2 mN/m at 22 °C, and stable for at least 2 h, resulting in a negligible amount of surface active impurities.

The cleaning of all glass and Teflon laboratory materials utilized in sample preparation was carried out by using sulphuric acid and then rinsing with Milli-Q water. Isopropanol was used for all other materials before rinsing with Milli-Q water.

### 4.2. Methods

#### 4.2.1. Interfacial Tension Measurement

Interfacial tension and dilational viscoelasticity were measured using a drop shape tensiometer (PAT1, Sinterface, Berlin, Germany), described in detail in [30]. For the present study, as in our previous study on the liquid–liquid interface [21], we used a configuration where an emerging drop of oil with a volume of about 30 μL is surrounded by the surfactant aqueous solution, minimizing, in this way, the possible depletion of the surfactant solution due to adsorption and transfer into the oil phase. During the interfacial tension acquisition, the drop of oil, after its rapid formation at the tip of a steel capillary, is maintained at constant area. In this way, the adsorption kinetics are monitored on an initially “fresh” interface until the achievement of the equilibrium.

To measure the dilational viscoelasticity E versus frequency, the PAT tensiometer was used in the same configuration according to the oscillating drop method [31]. According to this method, after the achievement of the adsorption equilibrium, a controlled area oscillation is imposed on the oil drop while the interfacial tension is measured.

The amplitude of the area perturbation was not higher than 2% and a frequency range from 0.005 to 0.2 Hz was maintained in order to ensure, respectively, the linear response of the interfacial layer and the Laplacian profile of the drop during oscillation.

The surface tension measurements necessary for selecting fluorescent dyes for confocal microscopy were conducted using a pendant drop shape method in a homemade apparatus, characterized by lower accuracy but appropriate for the required tests.

All the measurements reported in this work were executed at a controlled temperature of 20 ± 1 °C.

#### 4.2.2. Emulsion Production and Characterization

Emulsions were produced using a double-syringe (DS) method, as in our previous work on foams [19] and emulsions [21], where the method is described in detail. The formation of the emulsion is induced by the repetitive exchange of liquid between two syringes containing the two phases to be emulsified, connected by a short narrow pipe. For the present study, we used two 5 mL standard syringes connected through a pipe 15 mm long with an inner diameter of 1 mm. The water–oil volume ratio was one for all emulsions investigated (2.5 mL for each liquid phase), and the number of sequential pushing cycles was 20. As in [19,21], the choice of these latter parameters has been determined by the necessity of maximizing emulsification efficiency and, at the same time, obtaining marginally stable emulsions that are appropriate for comparative investigations.

After formation, emulsions inside the syringes are monitored for at least 2 days. Their height and the height of the separated phases are measured by using a ruler with a resolution of 0.5 mm.

To check the morphology of the obtained emulsions, a small amount of them (~0.1 mL) are taken from the syringe immediately after the formation, diluted 10 times with the aqueous solution, and placed between two glass slides separated by a spacer of 0.2 mm thickness to be analyzed by optical microscope. A reflected light DVM6-M microscope (Leica, Hamburg, Germany), equipped with the PlanAPO FOV 3.60 objective, was used, providing a resolution better than 1 micron.

#### 4.2.3. Confocal Microscopy

A deeper analysis of the emulsion structure was obtained using a Carl Zeiss LSM780 (Carl Zeiss, Jena, Germany) confocal microscope. For this analysis, emulsions are placed into an ad hoc-designed glass Petri dish immediately after their preparation. To increase the contrast and improve the visualization of the two liquid phases, Coumarin 6 and Rhodamine B were added to the oil and aqueous phases before the emulsification. The selection of these fluorescent dyes was performed on the basis of key factors, such as solubility in the involved liquid phases, efficiency in color change and, at the same time, the absence of perturbing effects on the adsorption interfacial layers and consequently on emulsion properties. Concerning this latter aspect, the possible surface activity of dyes at the water–oil interface was analyzed by interfacial tension measurements.

#### 4.2.4. Multiple Light Scattering Measurements

Emulsion evolution was also analyzed by turbidity measurements using a MultiScan MS 20 (DataPhysics Instruments GmbH, Filderstadt, Germany), supported by MSC control and evaluation software (https://www.dataphysics-instruments.com/us/products/ms/software, accessed on 15 March 2025).

According to the experimental set up sketched in Figure S9 of the Supplementary Materials, the Multiscan MS 20 records 0° transmission and 45° backscattering profiles. For this study, measurements were performed in a cell with a diameter of 10 mm and a height of 56 mm, using monochromic NIR radiation at the wavelength 870 nm for transmission and 470 nm (blue light) for backscattering. The lower-frequency beam is weakly scattered and tends to be absorbed, which is advantageous for measuring sample transparency. The higher-frequency beam is more scattered on small particles, thus measuring their concentration and size more precisely. The use of different wavelengths minimizes the possibility of interference between the transmitted and reflected light beams. Emulsions were generated by the method described above and injected into the experimental cell immediately before starting the measurements.

Backscattering and transmission intensities versus sample height and time were recorded, allowing the monitoring of the emulsion ageing. Due to the expected long duration of the emulsion destabilization, we tested it initially in a continuous mode, every 20 s for 5 min and later at selected times from the generation: 15, 30, 60, 90, 120, and 180 min.

It should be noted that both the aqueous solution of saponin and MCT oil are almost completely transparent to light radiation. The aqueous solution of saponin transmits light practically like water, and the oil does not differ in this respect by more than 15% (transmittance of light radiation larger than 85% in relation to the reference pure water). This results in the very low backscattering profiles of both liquids, at the level of 2% above the reference level of pure water. Thanks to this, all turbidity disorders related to the formation of emulsions of various types (water in oil, oil in water, and more complex multi-emulsified systems) can be easily detected.

Thus, as indicated in Figure S5, this analysis allows creaming or sedimentation processes to be detected during emulsion ageing and, these being strictly related to the coalescence of oil and water droplets, also enables an advancing hypothesis on the destabilising mechanisms and their dependence on the emulsion composition.

All experiments on emulsion stability and their formation have been performed at room temperature in a range of 20–22 °C.

#### 4.2.5. Molecular Dynamics Simulation

The molecular dynamic simulation was performed using YASARA Structure version 24.4 package [32]. The chitosan molecule was constructed of 28 glucosamine or N-acetyl glucosamine units, assuming 85% deacetylation. The degree of chitosan charging was determined by the protonation of the selected amine groups. The simplified saponin used in the simulation, consisting of glucuronic acid and aglycone [33,34] to assure anionic type surfactant behaviour, is illustrated in Figure 3. One chitosan chain and adjacent two saponins were placed in the simulation box with a size of 10.7·10.7·10.7 nm, filled with water molecules (TIP3P, density 1 g/L) and Na^+^ and Cl^−^ ions to retain the electroneutrality of the system. We used AMBER 14 force field [35] with GLYCAM [36] parameters for the chitosan. Other parameters (partial charges) were assigned using the AM1 semi-empirical calculation in the YASARA structure package. The simulation was run for 20 ns to equilibrate the system, then it was continued for 200 ns and the positions of the investigated molecules were monitored. The number of hydrogen bonds and the strength of hydrophobic interactions were calculated using algorithms implemented in the YASARA package.

## 5. Conclusions

In this work, we investigated the properties of saponin solutions in the presence of acetic acid and chitosan both from the points of view of adsorption at the water–oil interfaces and the capacity of oil-in-water emulsions in emulsification and stabilization. The study relies on different experimental approaches for the emulsion and interfacial layer characterization and is also complemented by molecular dynamics calculations. This multi-technique approach allowed us to advance the study of the thermodynamic and dynamic properties of the interfacial adsorbed layer at the water–oil interface and to put them in relation to the properties of the respective emulsions.

The results of the present work, with respect to those previously obtained with similar systems, enable advancements in the comprehension of the mechanisms at the bases of emulsion stabilization, which mainly consists of preventing droplet coalescence.

In particular, the formation of amphiphilic chitosan–saponin complexes, already hypothesized in previous works, is here confirmed. This study also offers new insights into the properties of the adsorbed layers at the surface of the oil droplets and a better understanding of the important role of such complexes in stabilization against droplet coalescence, paying particular attention to the effect of pH and to the onset, under certain conditions, of double emulsification.

In particular, in neutral conditions, the good stabilizing effect of saponin on MCT-in-water emulsions can be attributed to the electrostatic repulsion of the adsorbed layers because, in pure water, saponin is dissociated; however, this is not true when saponin is dissolved in an acid environment due to its non-ionic character. The improvement of the stability of oil in water emulsions, observed in the presence of chitosan, can be explained by considering the role of the amphiphilic saponin–chitosan complexes which, besides conferring positive charge to the interface, make the interfacial layers at the drop surfaces rather rigid so that emulsions are stabilized both for electrostatic repulsion and steric effects.

Finally, the interpretation of the interfacial properties results, in light of the formation of saponin–chitosan complexes with composition-dependent amphiphilicity, provides an explanation for the formation of double water-in-oil-in-water emulsions observed and for their dependence on the presence and concentration of chitosan.

## Figures and Tables

**Table 1:** Parameters by the analysis of the interfacial tension–concentration isotherm.

Interface	Γ_max_ (μ mol/m^2^) *	cmc (g/L)	cmc (M) *	γ_cmc_ (mN/m)
saponin solution/air	5.2 ± 0.1	0.65	3.9 × 10^−4^	39.1
saponin + acetic acid solution/air	7.8 ± 0.4	0.11	6.7 × 10^−5^	38.7
saponin solution/MCT	1.2 ± 0.1	0.79	4.8 × 10^−4^	7.3
saponin + acetic acid solution/MCT	0.9 ± 0.1	0.14	8.5 × 10^−5^	5.3

* Adsorption and bulk concentrations calculated assuming 1650 g/mol as the molar weight of saponin.

**Table 2:** Adsorption isotherm parameters by fitting the reorientation model to data reported in Figure 1c, assuming 1650 g/mol as the molar weight of saponin.

Aqueous Phase	ω_1_ (m^2^/mol)	ω_2_ (m^2^/mol)	α	b (m^3^/mol)
saponin solution	(1.20 ± 0.02) 10^6^	(3.60 ± 0.02) 10^5^	4.4 ± 0.1	15.2 ± 0.2
saponin + acetic acid solution	(1.20 ± 0.02) 10^6^	(1.40 ± 0.02) 10^5^	4.7 ± 0.1	3.7 ± 0.2

**Table 3:** Adsorption isotherm parameters by fitting the reorientation model to data reported in Figure 1c, assuming 1650 g/mol as the molar weight of saponin.

Chitosan Charge	Saponin Charge	Probability of Forming Aggregate	Hydrogen Bonds Energy (kJ/mol)
+24 e	0	<0.05 ± 0.1	-
+15 e	0	0.15 ± 0.1	8 ± 5
+7 e	0	0.35 ± 0.1	15 ± 5
+7 e	−1 e	0.55 ± 0.1	20 ± 5
+15 e	−1 e	>0.95 *	30 ± 8

* Adsorption and bulk concentrations calculated assuming 1650 g/mol as the molar weight of saponin.

## Data Availability

The data presented in this study are freely available from the authors upon a reasonable request.

## Supplementary Materials

The following supporting information can be downloaded at https://www.mdpi.com/article/10.3390/molecules30112281/s1, Table S1: Equilibrium values of surface tension with and without fluorescent dyes; Figure S1: Dynamic interfacial tension in two different configurations; Figure S2: Best fit parameters obtained using theoretical mod(E) expression; Figure S3: Time evolution during the MD simulation of the distance between weakly charged chitosan (+7 e) and saponin (−1 e) molecules; Figure S4: Time evolution during the MD simulation of the distance between weakly charged chitosan (+7 e) and uncharged saponin molecules; Figure S5: Backscattering (left) and transmission (right) intensity profiles acquired by Multiscan MS 20 (emulsions obtained with 0.3 g/L chitosan); Figure S6: Backscattering (left) and transmission (right) intensity profiles acquired by Multiscan MS 20 (emulsions obtained with 0.1 g/L chitosan); Figure S7: Photos of the emulsions in the Multiscan 20 cell, obtained with 0.3 g/L chitosan; Figure S8: Photos of the emulsions in the Multiscan 20 cell, obtained with 0.1 g/L chitosan; Figure S9: Setup for the stability analysis system.
