# Supplementary material for: Adsorption of Saponin and Saponin–Chitosan Mixture at Water–Oil Interface and Stabilization of Oil-in-Water Emulsions
Source: Molecules. 2025 May 22;30(11):2281. doi: 10.3390/molecules30112281 (PMC12156195; doi:10.3390/molecules30112281)
Supplement: Supplementary file 1 [file molecules-30-02281-s001.zip › molecules-3593027-supplementary.pdf]

Adsorption of saponin and saponin-chitosan mixture at water-MCT interface and stabilization of oil-in-water emulsions

Katarzyna Dziza[1], Marcel Krzan[2], Ewelina Jarek[2], Lilian Szyk-Warszyńska[2], Sonia Kudłacik-Kramarczyk[2], Piotr Warszyński[2], Eva Santini[1], Libero Liggieri[1], Francesca Ravera[1,*]

Supplementary Material

Effect of fluorescent dyes on the interfacial properties of liquid phases

Table S1: Equilibrium values of surface tension with and without fluorescent dyes

interface	Surface tension mN/m, T= 22°C	St. Dev.
air/water	72.3	0.6
air/1% acetic acid	69.5	0.7
air/rhodamine $3{\cdot}10^{-6}$ M	72.4	0.9
air/rhodamine $3{\cdot}10^{-6}$ M in 1% acetic acid	69.3	0.6
air/MCT	29.7	0.9
air/MCT coumarin 6	30.0	0.8
water/MCT	24.3	0.6
water/MCT coumarin 6	23.6	0.7
1% acetic acid/ MCT coumarin	21,5	0,6
rhodamine $3{\cdot}10^{-6}$M in water/ MCT coumarin 6	24.4	1.4
rhodamine $3{\cdot}10^{-6}$M in 1% acetic acid/ MCT coumarin 6	23.3	1

Partitioning of saponin among water and MCT

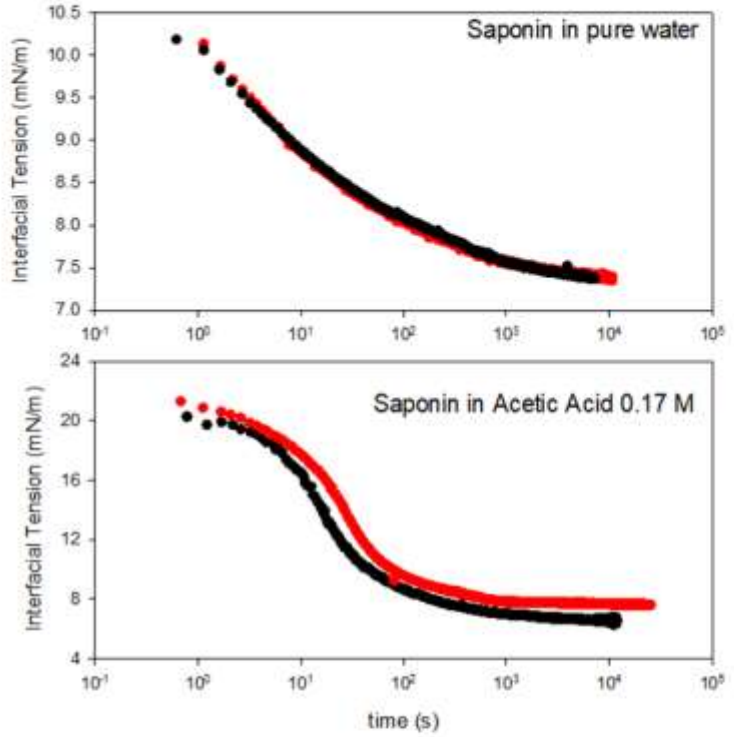

Figure S1: dynamic interfacial tension in two different configurations: emerging MCT drop in saponin solution (black) and pendant drop of solution in initially pure MCT (red) to check the transfer of saponin from the aqueous phase to oil. Saponin 0.8 g/l in pure water (above) and Saponin 0.08 g/l in acetic acid 0.17 M (below).

Results from dilational rheology

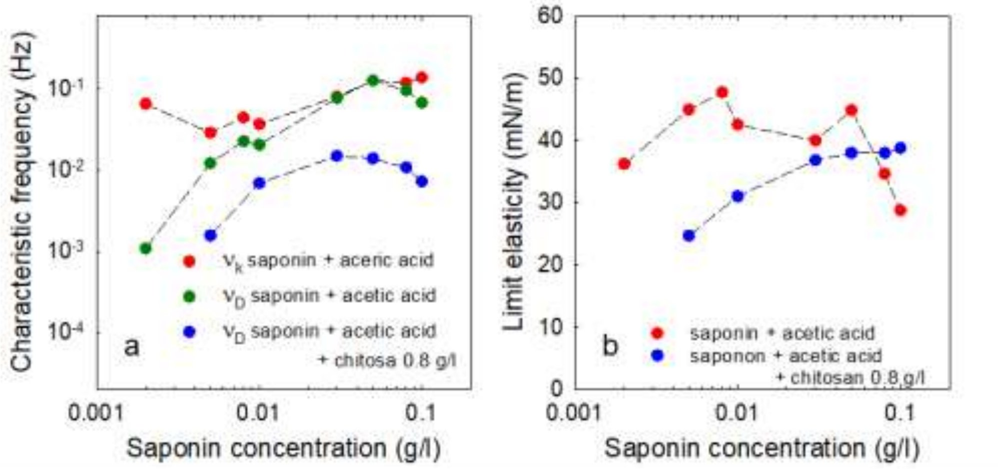

Figure S2: Best Fit parameters obtained using theoretical mod(E) expression calculated by Eq. 3 to experimental data reported in Figure 3a versus saponin concentration: characteristic frequencies of the kinetic interfacial process (re-orientation) and of diffusion controlled adsorption (a) and high frequency limits of the dilational elasticity (b).

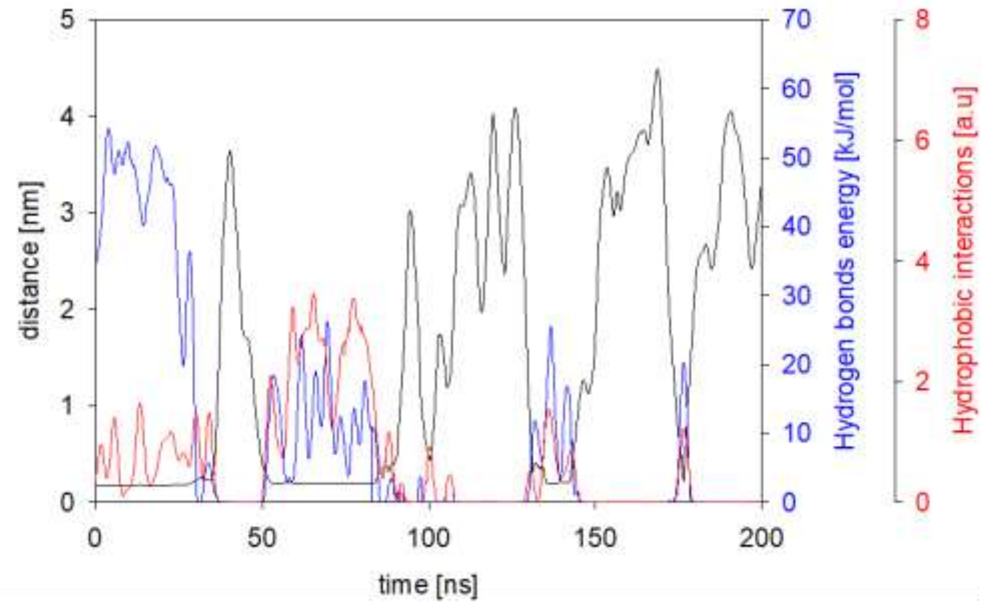

Figure S3: Time evolution during the MD simulation of the distance between weakly charged chitosan (+7e) and saponin (-1e) molecules (black line), hydrogen bonds energy (blue line) and hydrophobic interactions (red line).

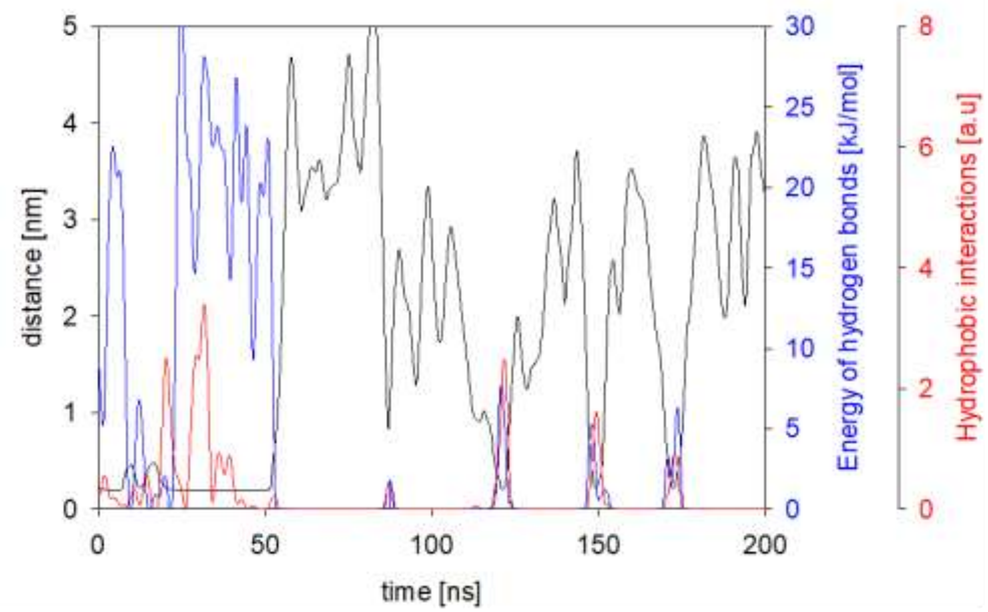

Figure S4: Time evolution during the MD simulation of the distance between weakly charged chitosan (+7e) and uncharged saponin molecules (black line), hydrogen bonds energy (blue line) and hydrophobic interactions (red line).

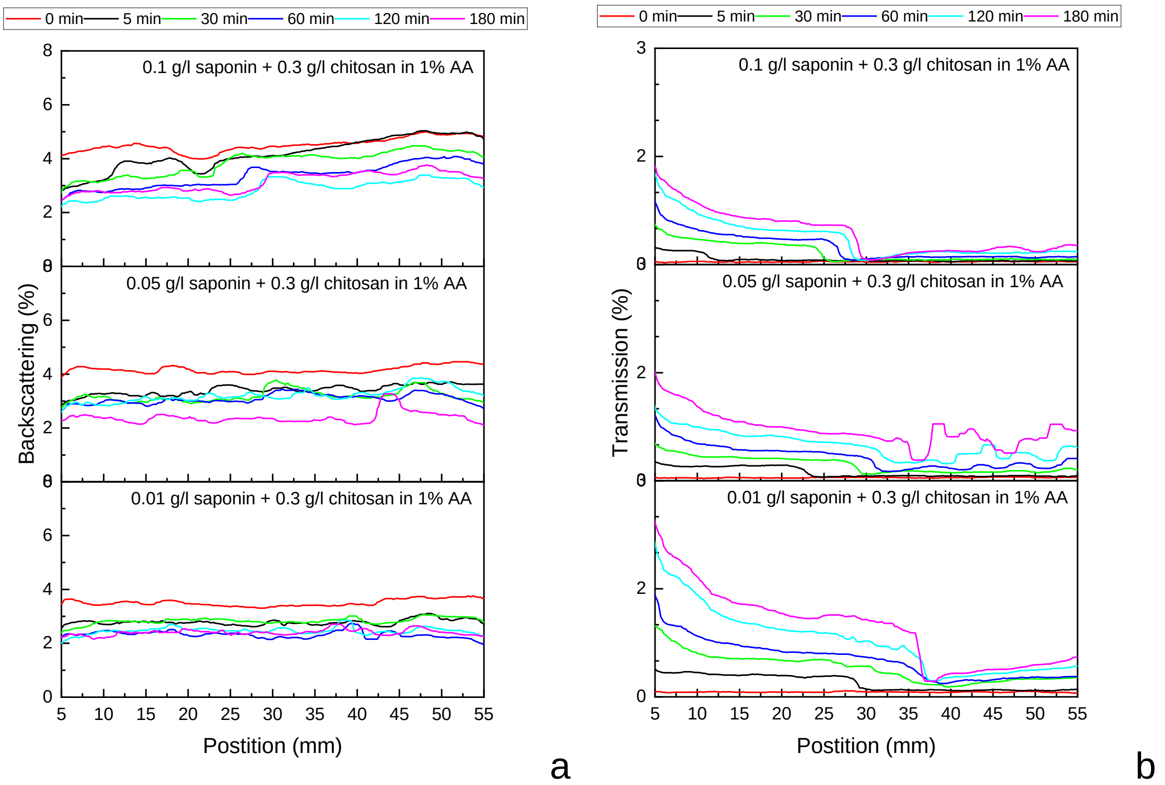

Figure S5: Backscattering (left) and transmission (right) intensity profiles acquired by Multiscan MS 20. Emulsions obtained with 0.3 g/l chitosan and 0.01g/l, 0.05g/l and 0.1g/l of saponin.

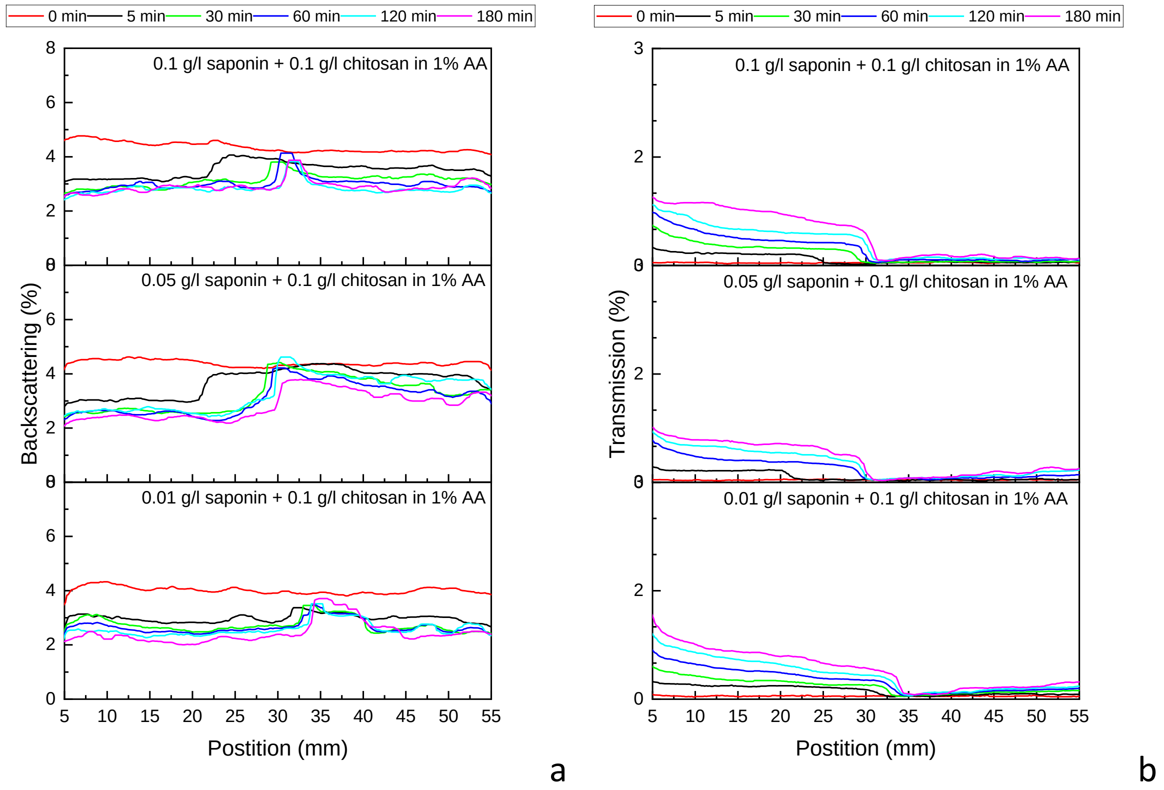

Figure S6: Backscattering (left) and transmission (right) intensity profiles acquired by Multiscan MS 20. Emulsions obtained with 0.1 g/l chitosan and 0.01g/l; 0.05g/l and 0.1g/l of saponin.

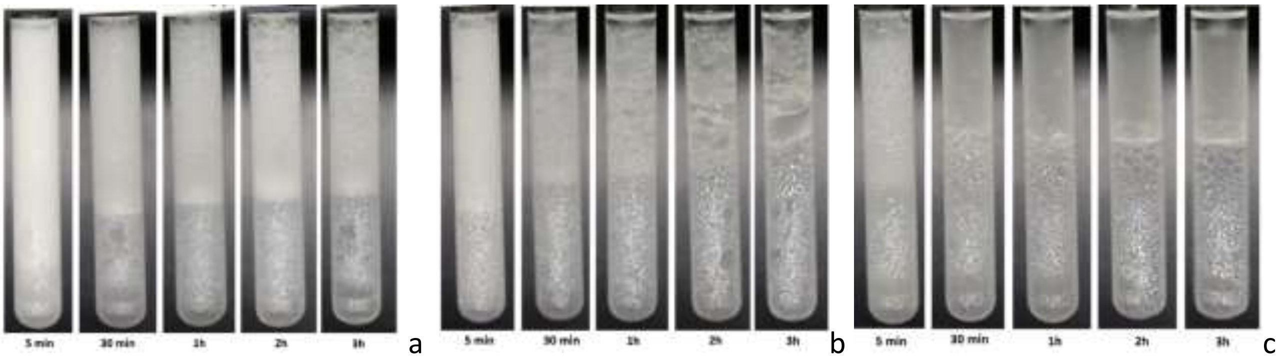

Figure S7: Photos of the emulsions in the Multiscan 20 cell. Emulsions obtained with 0.3 g/l chitosan and various saponin concentrations: A - 0.1g/l saponin; B - 0.05g/l saponin and C - 0.01g/l saponin.

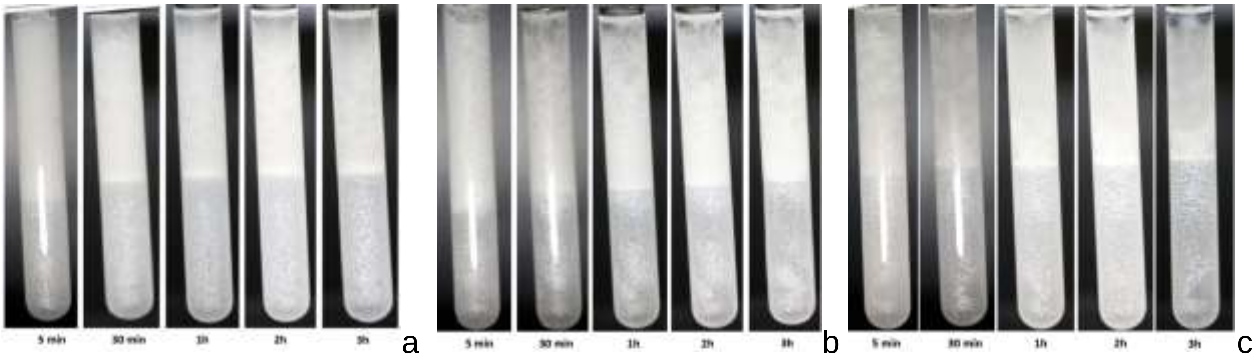

Figure S8: Photos of the emulsions in the Multiscan 20 cell. Emulsions obtained with 0.1 g/l chitosan and various saponin concentrations: A - 0.1g/l saponin; B - 0.05g/l saponin and C - 0.01g/l saponin

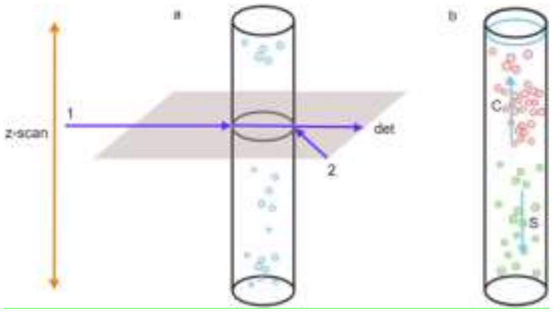

Figure S9: Setup for the stability analysis system: MultiScan MS 20 with two light sources (1 at 870 nm; 2 at 470 nm) and a) detector which repeatedly scans the sample along the z-axis; b) schematic representation of the recorded processes (C for creaming; S for sedimentation or cracking)
